# Detection of a heterozygous germline *APC* mutation in a three-generation family with familial adenomatous polyposis using targeted massive parallel sequencing in Vietnam

**DOI:** 10.1186/s12881-018-0701-y

**Published:** 2018-10-19

**Authors:** Hoa Giang, Vu T Nguyen, Sinh D Nguyen, Huu-Phuc Nguyen, Binh T Vo, Truc M Nguyen, Nguyen H Nguyen, Kiet D Truong, Thanh-Thuy T Do, Minh-Duy Phan, Hoai-Nghia Nguyen

**Affiliations:** 1 Center for Molecular Medicine, University of Medicine and Pharmacy, Ho Chi Minh city, Vietnam; 2 Gene Solutions, Ho Chi Minh city, Vietnam; 3 Department of Oncology, University of Medicine and Pharmacy, Ho Chi Minh city, Vietnam; 4 Ung Buou Hospital, Ho Chi Minh city, Vietnam; 5 Gia Dinh Hospital, Ho Chi Minh city, Vietnam; 6 Thu Duc Hospital, Ho Chi Minh city, Vietnam; 7 Medical Genetics Institute, Ho Chi Minh city, Vietnam; 8 Graduate Program of Genetics, University of Science, Ho Chi Minh city, Vietnam; 9 Vinmec Central Park International Hospital, Ho Chi Minh city, Vietnam

**Keywords:** Familial adenomatous polyposis, APC gene, Germline mutation, Genetic testing, Massively parallel sequencing

## Abstract

**Background:**

Familial adenomatous polyposis (FAP) is an autosomal dominant hereditary syndrome characterised by the development of hundreds to thousands of adenomatous colonic polyps during the second decade of life. FAP is caused by germ line mutations in the adenomatous polyposis coli (*APC*) gene located on chromosome 5q21–22.

**Case presentation:**

A 36-year-old female was presented with 100–1000 adenomatous colonic polyps, typical of classic FAP symptoms. Genetic testing using massively parallel sequencing identified a 5-bp deletion (c.3927_3931delAAAGA) which causes frameshift (p.Glu1309Aspfs) and creates a premature stop codon, resulting in the replacement of the last 1535 amino acids of *APC* by five incorrect amino acids. Two of the proband’s four siblings also exhibited classic FAP symptoms and carried the same 5-bp heterozygous deletion in the *APC* gene. One of the proband’s two nephews also tested positive for this mutation but has not been examined by endoscopy due to his young age.

**Conclusions:**

We reported here for the first time the use of massively parallel sequencing (MPS)-based genetic testing to identify a germline mutation within a three-generation Vietnamese family. This mutation is most likely responsible for the development of FAP.

## Background

Familial adenomatous polyposis (FAP) is an autosomal dominant hereditary syndrome characterised by the development of hundreds to thousands of adenomatous colonic polyps during the second decade of life. FAP has an estimated prevalence of once in every 10,000 births [1] and if left untreated, colon cancer is essentially inevitable. The mean age of colon cancer diagnosis is 39 years and 7% of untreated individuals develop cancer by age 21 and 90% by age 45 [2]. FAP is caused by germ line mutations in the adenomatous polyposis coli (*APC*) gene located on chromosome 5q21–22 [1]. Apart from FAP, mutations in *APC* are also associated with attenuated FAP, and gastric adenocarcinoma and proximal polyposis of the stomach (GAPPS). Currently there are more than 750 mutations in the *APC* gene that were classified as pathogenic or likely pathogenic in ClinVar database. The majority of disease-causing mutations identified to date are gene inactivating and result in APC protein truncation [1]. It is known that patients with identical mutations can develop different clinical features [3]. However, some correlations between the location of mutations in the *APC* gene and phenotype have been observed. For example, the average age of onset in individuals with colonic symptoms varied by mutation locations, with onset age of 20 years for mutations at codon 1309, age of 30 years for mutations between 168 and 1580 and age of 52 years for mutations at 5′ of codon 168 and 3′ of codon 1580 [4].

Individuals who have clinical diagnosis or clinical suspicion of FAP or who are at-risk family members or who have as few as 10–15 adenomatous polyps might benefit from genetic testings [5]. In recent years, genetic tests using massively parallel sequencing (MPS) technologies have become more affordable. However, the adoption rates of such MPS-based genetic tests in developing countries such as Vietnam remain very low. In this study, we report the use of MPS to identify a mutation in the *APC* gene that are linked with FAP in a family of three generations in Vietnam.

## Case presentation

### Clinical information

**Fig. 1:**
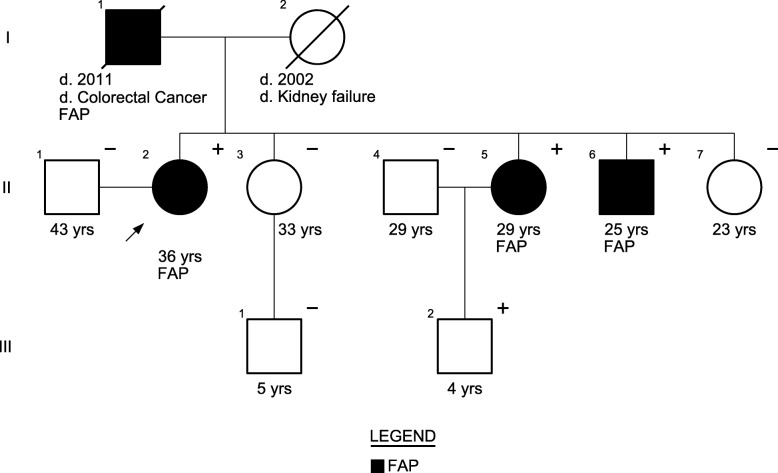
Pedigree of a three-generation family with familial adenomatous polyposis. Arrow points to the proband (II-2). Shading indicates family members that were diagnosed with FAP. Squares and circles denote males and females respectively. Roman number indicates generations. Plus and minus signs indicate presence of *APC* deletion

**Fig. 2:**
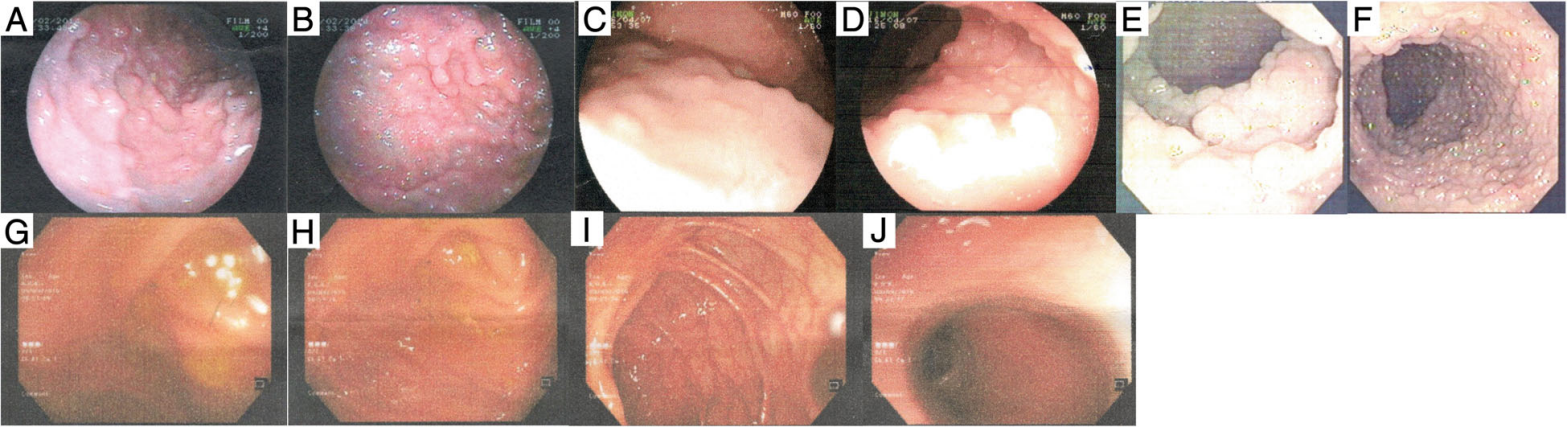
Colonoscopy analysis of affected and unaffected members. **a-f** Familial adenomatous polyp: multiple polyps in the colon of affected members (A-B: II-2, C-D: II-5, E-F: II-6, respectively). **g-j** Polyps were not observed in unaffected members (G-H: II-3, I-J: II7)

A 36-year-old female (proband, II-2, Fig. 1) had reported to the Department of Oncology, Thu Duc hospital (Ho Chi Minh City, Vietnam) with recurrence of frequent diarrhea and stool mixed with blood and mucus. Endoscopy revealed 100–1000 colonic polyps with the size in range of 5–15 mm (Fig. 2). Colonic polypectomy was later performed to prevent the development of colonic cancer. Family history investigation revealed that the proband’s father died of colorectal cancer at the age of 51 year. Endoscopic screenings were therefore performed on all of her siblings. Two of her four siblings (II-5 and II-6) also exhibited 100–1000 colonic polyps suggesting of FAP syndrome in this family (Fig. 2). These patients did not exhibited other non-colonic manifestations such as congenital hypertrophy of the retinal pigment epithelium (CHRPE) or desmoid tumors.

### Mutation analysis

To identify the genetic mutation(s) that might have caused the FAP in this family, a MPS-based *oncoSure* hereditary cancer test (Gene Solutions, Vietnam) was chosen to perform on all nine members of the family including the proband, her spouse, all of her siblings and their spouses as well as their offsprings. The *oncoSure* hereditary cancer test is a 17-gene panel including *BRCA1, BRCA2, PALB2, PTEN, TP53, CDH1, MLH1, MSH2, MSH6, PMS2, EPCAM, APC, MUTYH, STK11, VHL, RB1, RET* that identifies an elevated risk for 10 hereditary cancers: breast, ovarian, colorectal, endometrial, gastric, pancreatic, prostate, melanoma, endocrine and retinoblastoma. Blood samples were collected and genomic DNA were extracted with QiaAmp DNA blood mini kit from Qiagen (Hilden, Germany) following the manufacturer’s instructions. DNA fragmentation and library preparation were done using NEBNext Ultra II DNA Library Prep Kit from New England BioLabs (Ipswich, MA, USA). Pool of sequencing libraries was captured using predesigned probes for 17 target genes from IDTDNA (Coralville, IA, USA). The targeted sequences covered all exons and a small flanking sequence of introns. Captured products were amplified with KAPA HiFi HotStart ReadyMix from KAPA Biosystems (Wilmington, MA, USA). Samples were sequenced on Illumina MiniSeq platform (Illumina, San Diego, CA, USA). Raw sequences from each sample were aligned to the reference human genome from University of California, Santa Cruz (UCSC) Genome Browser (NCBI build GRCh38) using Burrows Wheeler Aligner (BWA) [6]. The aligned output was used to compute depth and breadth of coverage in the target region, and SNP/INDEL calling with GATK standard pipeline [7]. Variants were classified using ClinVar database (National Institutes of Health).

A heterozygous deletion c.3927_3931delAAAGA (p.Glu1309Aspfs) in the *APC* gene was found in the proband (II-2), all other affected members (II-5 and II-6) and a young male member in the third generation (4 years of age, III-2). No other pathogenic or likely pathogenic mutations was detected elsewhere in the genes targeted by the *oncoSure* hereditary cancer gene panel. This deletion was absent in all unaffected members (II-3 and II-7) and a 5 year-old third generation male member (III-1) (Table 1). We did not identify this mutation in the 50 normal control of the same ethnic origin and age range.

**Table 1:** Clinical and genetic characteristics of the affected and unaffected family members

ID	Sex	Present Age	Age at diagnosis	Colonscopy screening	*APC* mutation (c.3927_3931delAAAGA)
II-1	Male	43	NA	NA	No
II-2	Female	36	24	100–1000 colonic polyps (5–15 mm)	Yes
II-3	Female	33	24	< 10 colonic polyps	No
II-4	Male	29	NA	NA	No
II-5	Female	29	20	100–1000 colonic polyps (5–15 mm)	Yes
II-6	Male	25	18	100–1000 colonic polyps (5–15 mm)	Yes
II-7	Female	23	13	< 10 colonic polyps	No
III-1	Male	5	NA	NA	No
III-2	Male	4	NA	NA	Yes

This 5-bp deletion occurs at codon 1309 which causes frameshift and creates a premature stop codon at position 4 of the new reading frame, resulting in the replacement of the last 1535 amino acids of APC by three incorrect amino acids. This variant was predicted to cause loss of normal protein function through protein truncation. The truncated protein caused by this deletion is similar to that caused by another 5-bp deletion variant (NM_000038.5(APC):c.3920_3924delTAAAA) which was reported in two individuals for whom clinical APC testing was ordered (Evidence detailed provided by GeneDx in ClinVar).

### Confirmation of the novel mutation by sanger sequencing

**Fig. 3:**
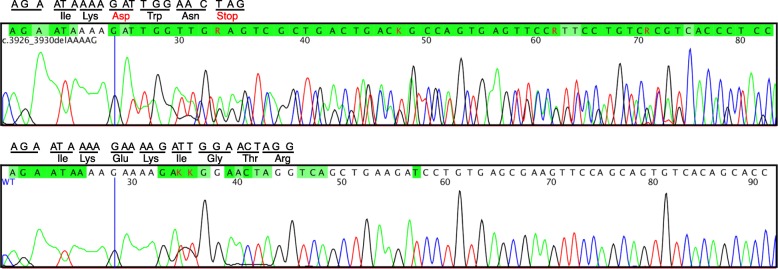
Validation of the deletion in affected members using Sanger sequencing. (Top) The heterozygous deletion c.3927_3931delAAAGA in proband II-2: the deletion causes a frameshift and creates a Stop codon downstream. (Bottom) The complete sequence in unaffected members. The blue vertical line represents the last base before deletion

To confirm this novel heterozygous deletion, we performed PCR and Sanger sequencing for all tested cases. We designed primers flanking the deletion location using the reference human genome from UCSC Genome Browser. The primers (5’-ATCAGACGACACAGGAAGCA-3′ and 5′- ACTCAGGCTGGATGAACAAGA-3′) were synthesized and purified by IDTDNA (Coralville, IA, USA). PCR amplification was prepared using Q5 High-Fidelity 2× Master Mix from New England BioLabs (Ipswich, MA, USA) following manufacturer’s instructions. The PCR products were sequenced using Applied Biosystems 3500xl. As shown in Fig. 3, the 5-nucleotide deletion was found in the proband but not in the unaffected member, confirming our finding using MPS technology.

## Discussion and conclusions

Familial adenomatous polyposis (FAP) is characterised by the development of hundreds to thousands of adenomatous colonic polyps during the second decade of life, and is a disease predisposing individuals to colorectal cancer (CRC) [1]. In this pedigree, the proband presented with rectal bleeding in combination with change in bowel habits, which was the most common symptom combination from the local tumour of CRC [8, 9], and endoscopic diagnosis showed symptoms of classic FAP (100–1000 polyps). A 5-bp heterozygous deletion c.3927_3931delAAAGA was detected in the *APC* gene, resulting in frameshift mutation (p.Glu1309Aspfs). The heterozygous mutation was suggestive of inheritance of this mutation from her father who had died of CRC. Unfortunately, we were unable to confirm the presence of this mutation in the proband’s father. Consistent with the theory that the proband inherited a wild-type *APC* allele from her mother and the mutant *APC* allele from her father, two of her four siblings (II-5 and II-6) also exhibited classic FAP symptoms and carried the same 5-bp heterozygous deletion in the *APC* gene. The proband’s nephew (III-2), son of her sister (II-5), also carried this heterozygous mutation but had not been examined by endoscopy due to his young age (4 years old). It was therefore crucial for the carriers in this family to have routine medical monitoring to properly manage and prevent the development of CRC. Any new offsprings from the carriers should also undertake genetic testings to determine the status of their *APC* alleles in order to have proper genetic counselling and early medical intervention.

FAP is caused by germ line mutations in the tumor suppressor gene, *Adenomatous Polyposis Coli* (*APC*), located on chromosome 5q21-q22 [1]. At the time of writing this report (Jun 2018), the ClinVar database [10] recorded 4471 variants in the *APC* gene, 609 of which were Pathogenic variants and 175 were Likely pathogenic. This c.3927_3931delAAAGA variant causes a frameshift (p.Glu1309Aspfs), results in a truncated version of *APC* gene. It falls between codons 169 to 1393, which are generally associated with classic FAP [3, 11] or more specifically, it falls in a region from codon 1250 to 1464 that is associated with severe FAP [12]. Furthermore, the truncation at codon 1309 has been shown to exert a dominant negative effect of *APC* gene products by inhibition of the wild-type APC activity [13]. The 100% correlation between the carriage of this mutation and FAP symptoms in all members over 20 years of age in our study further strengthens the interpretation of this variant as pathogenic. This mutation was first reported by Miyoshi et al. in a study searching for germline mutations of the *APC* gene in 79 unrelated FAP patients from the US and Japan [14]. This mutation was later reported as germline and somatic mutation in several other countries (Table 2). To the best of our knowledge, this is the first report using MPS to identify a variant in the *APC* gene in a Vietnamese pedigree.

**Table 2:** Reports of c.3927_3931delAAAGA mutation associated with FAP and/or colorectal tumour

Study	Year	Mutation type	Detection technology	Country
Miyoshi et al. [14]	1992	Germline	PCR-Sanger sequencing	US Japan
Cottrell et al. [15]	1992	Germline + Somatic	PCR-SSCP-Sanger sequencing	UK
Varesco et al. [16]	1993	Germline	PCR-SSCP-Sanger sequencing	Italy
De Benedetti et al. [17]	1994	Somatic	PCR-SSCP-Sanger sequencing	Italy
Yagi et al. [18]	1997	Somatic	PCR-SSCP-Sanger sequencing	Japan
Gryfe et al. [19]	1998	Somatic	PCR-Sanger sequencing	Canada
Sánchez-de-Abajo et al. [20]	2007	Somatic	PCR-Sanger sequencing	Spain
Syed Sameer et al. [21]	2011	Somatic	PCR-SSCP/ PCR-Sanger sequencing	India
Christie et al. [22]	2013	Somatic	PCR-Sanger sequencing	Australia
Chen et al. [23]	2015	Germline	MPS	China

The clinical application of MPS in the field of oncology has gained significant development and adoption in recent years. Mutation detection in hereditary cancer syndromes has major impact in genetic counselling, helps increase the chance of survival, defines the prognosis of carriers and identifies the most appropriate and personalised prophylactic measures [24]. In this study, we used a 17-gene panel (*oncoSure*) to detect germline mutations associated with 10 different hereditary cancer syndromes including Lynch syndrome, FAP and *MUTYH*-associated polyposis. Using this panel, we have successfully identified a mutation in the *APC* gene associated with FAP in a three-generation family. This mutation was verified by Sanger sequencing on all affected family members, validating our targeted MPS tests. MPS-based oncology research and clinical genetic testings are still relatively new in Vietnam and thus there is little information about the variants specific to Vietnamese population. This study is the first effort to report cases of cancer-associated germline variants found in the Vietnamese population. However, continuous surveillance using MPS technology is needed to build a comprehensive catalogue of pathogenic variants specific to Vietnam.

In conclusion, we reported a heterozygous mutation in the *APC* gene that was found in multiple members of a three-generation family in Vietnam. This mutation is most likely responsible for development of the FAP symptoms in carriers. We have deposited the information of this novel mutation to ClinVar (Variation ID: 816). To our knowledge, this is the first study in Vietnam that used MPS for the identification of cancer linked mutations.

## Abbreviations

APC: Adenomatous polyposis coli
CRC: Colorectal cancer
FAP: Familial adenomatous polyposis
MPS: Massive parallel sequencing
PCR: Polymerase chain reaction
SSCP: Single-strand conformation polymorphism
